# Exploring rural‐urban differences in modifiable dementia risk factors and cognitive performance in older Australians

**DOI:** 10.1002/alz70860_100636

**Published:** 2025-12-23

**Authors:** Britt Burton, Maddison L Mellow, Isabella Bower, Helen Barrie, Hannah Keage, Ashleigh E Smith

**Affiliations:** ^1^ Alliance for Research in Exercise, Nutrition and Activity (ARENA) Research Centre, University of South Australia, Adelaide, SA, Australia; ^2^ Cognitive Ageing and Impairment Neurosciences Laboratory (CAIN), University of South Australia, Adelaide, SA, Australia; ^3^ The Australian Alliance for Social Enterprise (TAASE), University of South Australia, Adelaide, SA, Australia

## Abstract

**Background:**

Despite growing evidence that dementia risk can be reduced through prevention strategies, rural populations often face unique challenges that may increase their vulnerability. However, the extent to which modifiable dementia risk factors vary between rural and urban communities – and their influence on cognitive performance – remains unclear. This cross‐sectional study examines differences in modifiable dementia risk factors and cognitive performance in older adults in rural and urban areas of Australia.

**Method:**

We analysed preliminary data from 236 older Australian adults (56 rural, 69% female; 180 urban, 81% female), with a mean age of approximately 68 years, as part of a larger, ongoing cohort study. Dementia risk was assessed using the total risk score from the Australian National University Alzheimer's Disease Risk Index (ANU‐ADRI), while cognitive function was measured using Addenbrooke's Cognitive Examination III (ACE‐III). Rural‐urban classification was determined using the Modified Monash Model.

**Results:**

T‐tests and Fisher's exact tests were used to compare modifiable dementia risk factors between urban and rural participants, revealing significant differences in cognitive performance and dementia risk. Urban residents scored higher on the ACE‐III than rural residents (M = 94.94 vs. 92.52; t(229) = ‐4.09; *p* < .001), and had lower dementia risk (M = ‐5.45 vs. ‐0.58; t(231) = 4.10; *p* < .001). Urban participants also had higher educational attainment (median 17 vs. 15 years, *p* < .001), engaged in more cognitive activities (median 3.18 vs. 3.00, *p* = .004), and consumed more fish (median 1.00 vs. 0.62, *p* = .006). Higher dementia risk, as indicated by ANU‐ADRI scores, was associated with lower cognitive performance (β = ‐0.13, *p* = .015), independent of rurality.

**Conclusion:**

These findings highlight important rural‐urban differences in modifiable dementia risk factors and cognitive performance among older Australians. The consistent relationship between risk factors and cognitive performance across settings suggests that prevention strategies may be equally effective in both rural and urban areas. However, the higher dementia risk score observed in rural populations underscores the need for targeted interventions. Future research will explore the underlying factors contributing to these differences and the feasibility of tailored interventions for rural communities.

